# Selective peripheral nerve recording using simulated human median nerve activity and convolutional neural networks

**DOI:** 10.1186/s12938-023-01181-0

**Published:** 2023-12-07

**Authors:** Taseen Jawad, Ryan G. L. Koh, José Zariffa

**Affiliations:** 1 KITE Research Institute, Toronto Rehabilitation Institute-University Health Network, Toronto, Canada; 2 Institute of Biomedical Engineering, University of Toronto, Toronto, Canada; 3 Edward S. Rogers Sr. Department of Electrical and Computer Engineering, University of Toronto, Toronto, Canada; 4 Rehabilitation Sciences Institute, University of Toronto, Toronto, Canada

## Abstract

**Background:**

It is difficult to create intuitive methods of controlling prosthetic limbs, often resulting in abandonment. Peripheral nerve interfaces can be used to convert motor intent into commands to a prosthesis. The Extraneural Spatiotemporal Compound Action Potentials Extraction Network (ESCAPE-NET) is a convolutional neural network (CNN) that has previously been demonstrated to be effective at discriminating neural sources in rat sciatic nerves. ESCAPE-NET was designed to operate using data from multi-channel nerve cuff arrays, and use the resulting spatiotemporal signatures to classify individual naturally evoked compound action potentials (nCAPs) based on differing source fascicles. The applicability of this approach to larger and more complex nerves is not well understood. To support future translation to humans, the objective of this study was to characterize the performance of this approach in a computational model of the human median nerve.

**Methods:**

Using a cross-sectional immunohistochemistry image of a human median nerve, a finite-element model was generated and used to simulate extraneural recordings. ESCAPE-NET was used to classify nCAPs based on source location, for varying numbers of sources and noise levels. The performance of ESCAPE-NET was also compared to ResNet-50 and MobileNet-V2 in the context of classifying human nerve cuff data.

**Results:**

Classification accuracy was found to be inversely related to the number of nCAP sources in ESCAPE-NET (3-class: 97.8% ± 0.1%; 10-class: 89.3% ± 5.4% in low-noise conditions, 3-class: 70.3% ± 0.1%; 10-class: 52.5% ± 0.3% in high-noise conditions). ESCAPE-NET overall outperformed both MobileNet-V2 (3-class: 96.5% ± 1.1%; 10-class: 84.9% ± 1.7% in low-noise conditions, 3-class: 86.0% ± 0.6%; 10-class: 41.4% ± 0.9% in high-noise conditions) and ResNet-50 (3-class: 71.2% ± 18.6%; 10-class: 40.1% ± 22.5% in low-noise conditions, 3-class: 81.3% ± 4.4%; 10-class: 31.9% ± 4.4% in high-noise conditions).

**Conclusion:**

All three networks were found to learn to differentiate nCAPs from different sources, as evidenced by performance levels well above chance in all cases. ESCAPE-NET was found to have the most robust performance, despite decreasing performance as the number of classes increased, and as noise was varied. These results provide valuable translational guidelines for designing neural interfaces for human use.

## Introduction

Every year, there are cases of injury or disease that result in the permanent loss of limb use. In 2005, there were an estimated 1.6 million people living with limb loss in the United States of America and that the number is estimated to increase to 3.6 million by 2050 [1–3]. The loss of a peripheral limb can result in significant reduction in quality of life, and currently, there is a need for improvements in post-amputation options for affected individuals [1–3]. While restoring any limb functionality can play a significant role in the independence of a person, hand function is integral to the human experience of interacting with the world. Robotic prostheses have matured to allow for more complex limb movements, but consequently require more sophisticated strategies for the user to translate their intent into motion. One potential source for data that can be used as a control signal for prosthetic limbs is recordings from peripheral nerves [4, 5], which offer the benefits of fully implanted systems and, unlike electromyography, can be applicable if substantial amounts of musculature are missing. Detailed control signals are of particular interest for the control of hand function, which is remarkable in its complexity.

Fascicles in peripheral nerves progressively branch off to innervate different motor units and sensory organs [6]. Therefore, by determining where an electroneurographic (ENG) signal originates inside a nerve, recordings can be associated with particular functions of interest and thus used to control assistive technologies [7–9]. The process of discriminating the bioelectric activity of multiple neural pathways within a peripheral nerve is referred to as selective recording [10].

While all peripheral nerve interfaces (PNI) are invasive in the sense that they are implanted devices, they vary in their degree of invasiveness. Extraneural, intraneural, and regenerative electrode arrays have all demonstrated capabilities for selective recording [4, 11, 12], but extraneural electrodes occasion the least amount of damage to the neural tissue and have been used for long-term implantations in humans [13]. Increasing the recording selectivity of extraneural PNIs, therefore, has important translations implications.

Previous work proposed a deep learning-based approach to increase the amount of information that could be extracted from extraneural recordings. The Extraneural Spatiotemporal Compound Action Potentials Extraction (ESCAPE) framework classifies individual naturally evoked compound action potentials (nCAPS) according to the neural pathway in which they originated. The classification is accomplished using a convolutional network (CNN) that is applied to spatiotemporal signatures derived from two-dimensional arrays of contacts in a nerve cuff electrode [14]. The CNN originally used in the ESCAPE framework is known as ESCAPE-NET. ESCAPE-NET has been evaluated previously on a rat sciatic nerve model, both in vivo and in simulation [9, 14–16]. While the framework was shown to be effective at classifying rat sciatic nerve data, its ability to scale up to larger and more complex nerves is not well understood.

To characterize the performance of ESCAPE-NET in nerve models more relevant to human applications and thus support translational efforts, this study focused on investigating the efficacy of the ESCAPE framework in a simulated human median nerve model. Specifically, the goal of was to characterize the ability of the ESCAPE selective recording framework to scale and classify increasing numbers of neural sources. To this end, two specific aims were formulated:

1. Characterize the relationship between the number of neural pathways to distinguish and the classification performance of ESCAPE-NET in a computational model of the human median nerve.
2. Investigate alternative neural network architectures to maximize the ability of the CNN to distinguish larger numbers of classes.

## Results

The steps used in this study were as follows, and are each detailed in the Methods section:

1. Using a cross-sectional image from a cadaveric sample, a finite-element (FE) model was created to represent a human median nerve segment.
2. Simulated nCAPs were generated using the FE model, using varying noise levels and source neural pathways within the nerve to create a physiologically representative dataset.
3. The simulated nCAP data were used to train ESCAPE-NET, ResNet-50, and MobileNet-V2, to evaluate the relationship between classification performance and complexity for different architectures by varying levels of noise and number of classes (i.e., number of potential source neural pathways).

### ESCAPE-NET

**Fig. 1:**
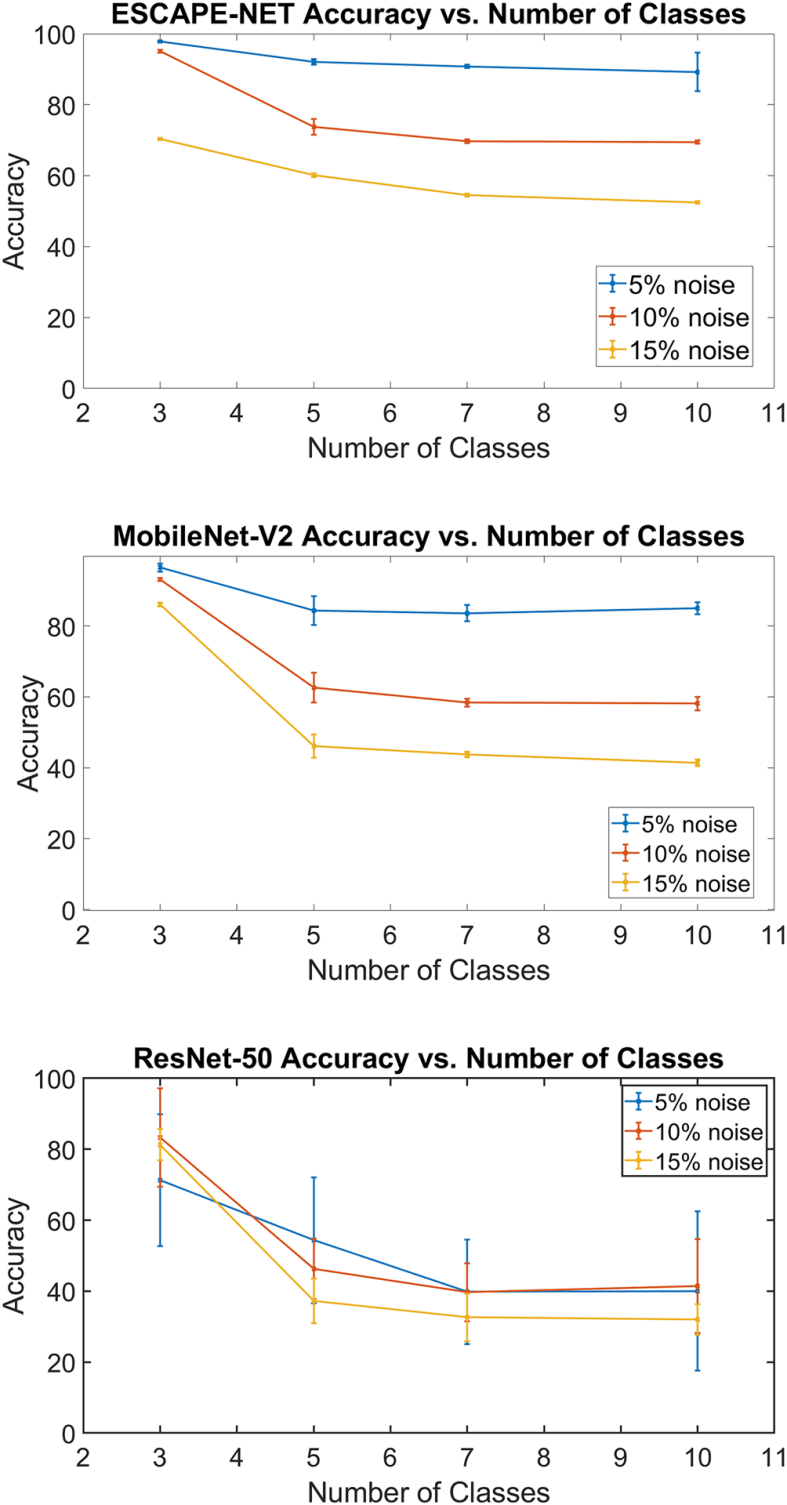
Accuracy vs. number of classes. ESCAPE-NET (Top), MobileNet-V2 (middle), and ResNet-50 (bottom)

ESCAPE-NET was found to have the highest accuracy and the lowest standard deviation. The accuracy was found to be inversely related with an increase in classes as well as an increase in the amount of noise (Fig. 1). The two-way ANOVA revealed a significant interaction between the effects of the number of classes and noise (*F*(6, 108)  = 1237.44, *p* < 0.001). Simple main effects analysis showed that noise levels had a significant effect on the classification accuracy (*p* < 0.001). Simple main effects analysis also showed a significant effect between the number of classes and the classification accuracy (*p* =  < 0.001).

When examining the variations as a function of the number of classes, the accuracy of the network had an average decrease from 97.8% ± 0.1% accuracy to 89.3% ± 5.49% in the 5% Noise case, and a more accentuated mean decrease in accuracy from 70.3% ± 0.1% to 52.4% ± 0.3% in the 15% noise case.

There was a measurable variation in the accuracy as the noise levels were varied. The noise levels tested in this study correspond to the maximum seen in vivo after filtering (15% of p–p signal amplitude). The mean classification accuracy change between 5% and 10% noise (mean: − 19.3% ± 1.0%) was a similar reduction as between 10% and 15% noise (mean: − 13.7% ± 0.7%).

The results suggest that ESCAPE-NET is a strong candidate for future studies involving the classification of in vivo human PNI data, if the number of sources required for the application is relatively low and signal quality is high.

### ResNet-50

ResNet-50 was found to have, on average, a lower classification accuracy when compared to ESCAPE-NET on the same conditions (Fig. 1). Similar to the previous experiment, there was a decrease in accuracy as the number of classes increased; however, the average accuracies were found to be lower and the variance wider. The 2-way ANOVA showed a significant interaction between the effects of the number of classes and noise (*F*(6,108) = 3.34, *p* < 0.01). Simple main effects analysis showed that noise levels had a significant effect on the classification accuracy (*p* < 0.01). Simple main effects analysis also showed a significant effect between the number of classes and the classification accuracy (*p* < 0.01).

It was hypothesized that ResNet-50 would outperform ESCAPE-NET; however, it was found that the overfitting of the network to the datasets resulted in lower accuracies when compared to ESCAPE-NET. The accuracy of the network had an average decrease from 71.2% ± 18.6% accuracy to 40.0% ± 22.4% in the 5% noise case, and a more accentuated mean decrease in accuracy from 81.3% ± 4.3% to 32.0% ± 4.4% in the 15% noise case.

ResNet-50 also exhibited the expected inverse correlation with an increase in the noise level in the generated data. The mean drop in classification accuracy between 5 and 10% noise (− 1.3% ± 6.8%) was found to be comparable to the mean drop between 10 and 15% noise (− 7.0% ± 3.9%).

### MobileNet-V2

MobileNetV2 was found to be similar in stability to ESCAPE-NET, albeit with slightly lower accuracies, and outperformed ResNet-50 (Fig. 1). The two-way ANOVA found a significant interaction between the effects of the number of classes and noise (*F*(6,108) = 144.44, *p* < 0.001). Simple main effects analysis showed that noise levels had a significant effect on the classification accuracy (*p* < 0.001). Simple main effects analysis also showed a significant effect between the number of classes and the classification accuracy (*p* < 0.001).

The negative relationship between accuracy and number of classes (as well as noise) decreased at similar rates as ESCAPE-NET and remained within 10% between these two networks. When selecting a network to perform classification during in vivo experiments, MobileNet-V2 may be a strong candidate of interest, considering its much smaller number of parameters.

When examining the variations as a function of the number of classes, the accuracy of the network had an average decrease from 96.4% ± 1.1% accuracy to 85.0% ± 1.7% in the 5% noise case, and a more accentuated mean decrease in accuracy from 86.0% ± 0.6% to 41.4% ± 0.9% in the 15% noise case.

Likewise, MobileNet-V2’s classification accuracy displayed the expected decrease as noise levels in the data increased, but nonetheless the network was still able to learn and perform classification well above chance levels. The mean drop in classification accuracy between 5 and 10% noise (mean: − 15.5% ± 0.7%) was similar to between 10 and 15% noise (mean: − 17.7 ± 0.3).

## Discussion

### Scalability to human nerves

The objective of this study was to determine if selective recording methods previously validated in an animal model could scale to a larger and more complex human nerve, the median nerve. The ESCAPE framework was found to be effective in selectively recording from human median nerves despite the more complex anatomy. One reason for these findings may be due to the diameter of the human nerve in this study (8 mm) being larger when compared to rat sciatic nerves (< 2 mm). This allowed for larger inter-contact distances on the electrode and allowed for lower levels of overlap across contact recordings, allowing for greater spatial selectivity. ESCAPE-NET had a range of 70–97% accuracy for the 3-class case across all noise levels and 50–90% accuracy for the 10-class case across all noise levels. For context, the original ESCAPE-NET evaluated on rat sciatic nerve data with 3 classes had an accuracy of 80.8% ± 10.4% and F1-score of 0.747 ± 0.114 [14]. The updated version of the CNN described in [15] and used in this study, when evaluated on that same dataset, had an accuracy of 80.1% ± 11.1% and F1-score of 0.721 ± 0.120 (unpublished). Based on the points above, it was determined that multi-channel extraneural signals combined with CNNs are a promising method of selectively recording nCAPs in human nerves.

The performance trends outlined in this study can be used when determining design parameters for future human studies. Both the evaluation of the impacts of increasing anatomical complexity and performance in varying noise conditions can be used to inform key requirements for future studies and implementations. To be applicable in vivo to human nerves, it is key to define the minimum requirements of the methodology in terms of accuracy, granularity in terms of classification (number of classes able to be classified accurately), as well as the maximum noise level that still produces good performance. In terms of the applicability of the modeling results, previous studies by our group provide context for the comparison of results from peripheral nerve FEMs and those from in vivo studies [14, 16]. It was found that there tended to be a reduction in accuracy of approximately 10% translating from modeling studies to in vivo application in the rat sciatic nerve. It is likely there can be a similar expected decrease of performance when planning a future in vivo study in humans. With this in mind, this study can inform future study designs by providing guidelines about expected performance for different neural networks as a function of classification task and noise levels.

### Assumptions and limitations

#### Modeling simplifications

A key simplification that was made in modeling is that a single immunohistochemistry (IHC) slice was extruded to form a uniform nerve segment (no variation in anatomy along the length of the nerve). This simplification was necessary due to the gaps in imaging of the fascicles throughout the length of the 5 cm, resulting from limitations of the imaging hardware and requirement to section the nerve into smaller segments. This implication of this simplification is that the interior of the nerve is more uniform than seen in vivo. In future studies, it is recommended that alternative imaging approaches, for example Micro-CT [17, 18], be used to enable the capture of the entirety of a tissue sample several centimeters in length.

#### Model variability

Future studies may also benefit from investigating different electrode dimensions and layouts. The Flat Interface Nerve Electrode (FINE) was chosen for this model due to its suitability for applications where the nerve segment has a flatter cross section. Additionally, a 56-channel contact arrangement was chosen for consistency with the previous studies that employed ESCAPE-NET [14, 16]. Electrodes with lower (or higher) spatial or temporal resolution warrant future investigation to determine appropriate trade-offs between instrumentation and algorithm complexity for a given application. It is possible further optimization of the networks may be required with different electrode designs. Longer electrodes (axially along the nerve) may provide greater temporal resolution, at the cost of requiring a larger segment of nerve to be accessible. If a thicker nerve segment is available, electrodes with greater inter-contact spaces circumferentially may allow for more identifiable spatial features and allow for greater classification accuracies. Conversely, smaller electrodes, both circumferentially and axially, or those with fewer channels may result in less informative features for the classification task and may require modifications and hyperparameter tuning of the networks to perform effective selective recording.

#### Noise simplification

The noise added to the data to simulate what is seen in vivo was white noise added at the amplitudes seen post-processing during in vivo experiments. The range chosen was representative of experimental observations; however, the noise model used is independent of the nerve geometry. The noise model assumed that noise from electronic and other bioelectric sources can be approximated as Gaussian white noise, which is reasonable given that additive noise from multiple sources will tend to a Gaussian distribution by the central limit theorem. However, more detailed noise models (e.g., similar to [19]) may improve the accuracy of the simulations in predicting in vivo performance. The impact of electromyographic artifacts that are not independent across contacts could also be investigated, as well as those of more transient effects such as movement artifacts. Reference montages other than a tripole configuration may also have an impact on the performance of the classification algorithms.

Furthermore, because of the greater distance between the contacts and active fascicles (on average) due to a larger nerve diameter, it is likely that the signal-to-noise ratio (SNR) would be lower in a human implant when compared to a rat experiment. It is recommended that, in future studies involving simulation, a more detailed noise model be used to encompass a wider range of use cases. Nonetheless, the range of noise values investigated in this study clearly illustrates the impact of noise on selective recording performance as the number of classes increases.

#### Overlapping nCAPs

A simplification is the presence of a single nCAPs at any one time in the time-series data. Cases of overlapping nCAP waveforms were not considered. The likelihood of overlaps occurring increases with the number of sources in the nerve. The impact of partial overlaps on the classification performance should be investigated in the future.

#### Fascicle distance

Each experimental case consisted of ten datasets and, due to the random nature of fascicle selection, the datasets contained a variety of fascicle spreads. Datasets consisted of fascicles next to each other as well as those geometrically far apart. In a future study, investigating the impact of fascicle distance may provide insights on other strengths and weaknesses of the ESCAPE framework and provide insight into potential modifications that improve the framework’s efficacy in in vivo situations and for clinical application. The effect of fascicle distance was evaluated here as a potential source of impact on classification accuracy; however, it was found that there was no correlation between fascicular distance and classification accuracy. The classification accuracy of ESCAPE-NET for all repetitions had < 1% variance, despite the different randomly selected combinations of fascicles with varying distances. This result was potentially due to the spatial distribution provided by a flatter cross-sectional area, the use of FINE, and the simulation of a 56 contact electrode. The cross-sectional fascicular distance may impact the performance of networks applied to electrodes with fewer channels or to locations with a more circular cross-sectional profile.

### Network comparisons

The smallest network (based on number of trainable parameters) investigated was MobileNet-V2 (3.4 million trainable parameters), followed by ResNet-50 (23 million parameters) followed by the largest ESCAPE-NET (92 million trainable parameters). The network that showed the best performance was ESCAPE-Net followed by MobileNet-V2 and then ResNet-50.

While MobileNet-V2 and ESCAPE-NET showed similar performance, the default ResNet-50 implementation was found to have diverging training and validation accuracies, suggesting the network was overfitting. This suggests that the network may be too complex for the task and is memorizing the data instead of learning the desired patterns. The variance in the results was the highest for ResNet-50, further suggesting overfitting. In future research, however, additional versions of ResNet can be investigated to explore possibilities and improvements in results that come from optimizing the parameter space or adding regularization. Regularization is a method of reducing the likelihood of a network overfitting the dataset by penalizing complexity, in this case using dropout layers to force the network to be able to operate with fewer weights. The 2022 version of ESCAPE-NET consisted of an architecture with built-in regularization in the form of dropout layers, which were potentially a factor in its stability and resistance to overfitting. The relatively small MobileNet-V2 network, at 3.4 million parameters, performed well without additional regularization.

All 3 networks saw similar decreases in performance due to increasing the number of classes; however, ESCAPE-NET and MobileNet-V2 were found to be more stable in comparison to ResNet-50—which had a higher variation in validation accuracy in each of the test cases. As the number of active fascicles increased from 3 to 10, ESCAPE-NET’s classification accuracy decreased by approximately 10–20%, depending on the noise level. While the performance obtained for high-noise levels was well above chance levels, it may not be sufficient to enable reliable neuroprosthetic control in practice. On the other hand, at low-noise levels, performance remained high even up to 10 classes. This suggests that pre-processing strategies are an important avenue for the practical application of the ESCAPE framework. It is also worth noting that MobileNet-V2 outperformed ESCAPE-NET for the 3-class case in 15% noise, and thus may be a good candidate when signal quality is low, but a limited number of control signals is acceptable. Overall, high performance could be achieved in the presence of a high number of classes or high-noise level, but overcoming both of these factors simultaneously proved challenging.

## Conclusion

The findings of this computational modeling study demonstrate that CNNs applied to multi-channel extraneural recordings are viable methods of selectively recording nCAPs in a human nerve, though significant decreases in performance can be expected when discriminating five or more distinct neural sources in noisy signals. ESCAPE-NET was found to have the most robust performance in comparison to MobileNet-V2 and ResNet-50. Based on these results, it can be concluded that the ESCAPE framework is a promising method for selectively recording in nerve anatomies more complex than the rat sciatic nerve and is a good candidate for classifying human extraneural ENG data.

The significance of this research comes threefold. The primary focus of this study was to provide a starting point for evaluating the scalability of the ESCAPE framework in a human model. A secondary benefit was the insights gained through the comparison of ESCAPE-NET with ResNet-50 and MobileNet-V2 within the context of classification of spatiotemporal signatures. Finally, an additional benefit of this study was the development of an FEM model of the human median nerve, which can serve as a platform for further experiments to support translation of these approaches to humans.

## Methods

### Human median nerve

**Fig. 2:**
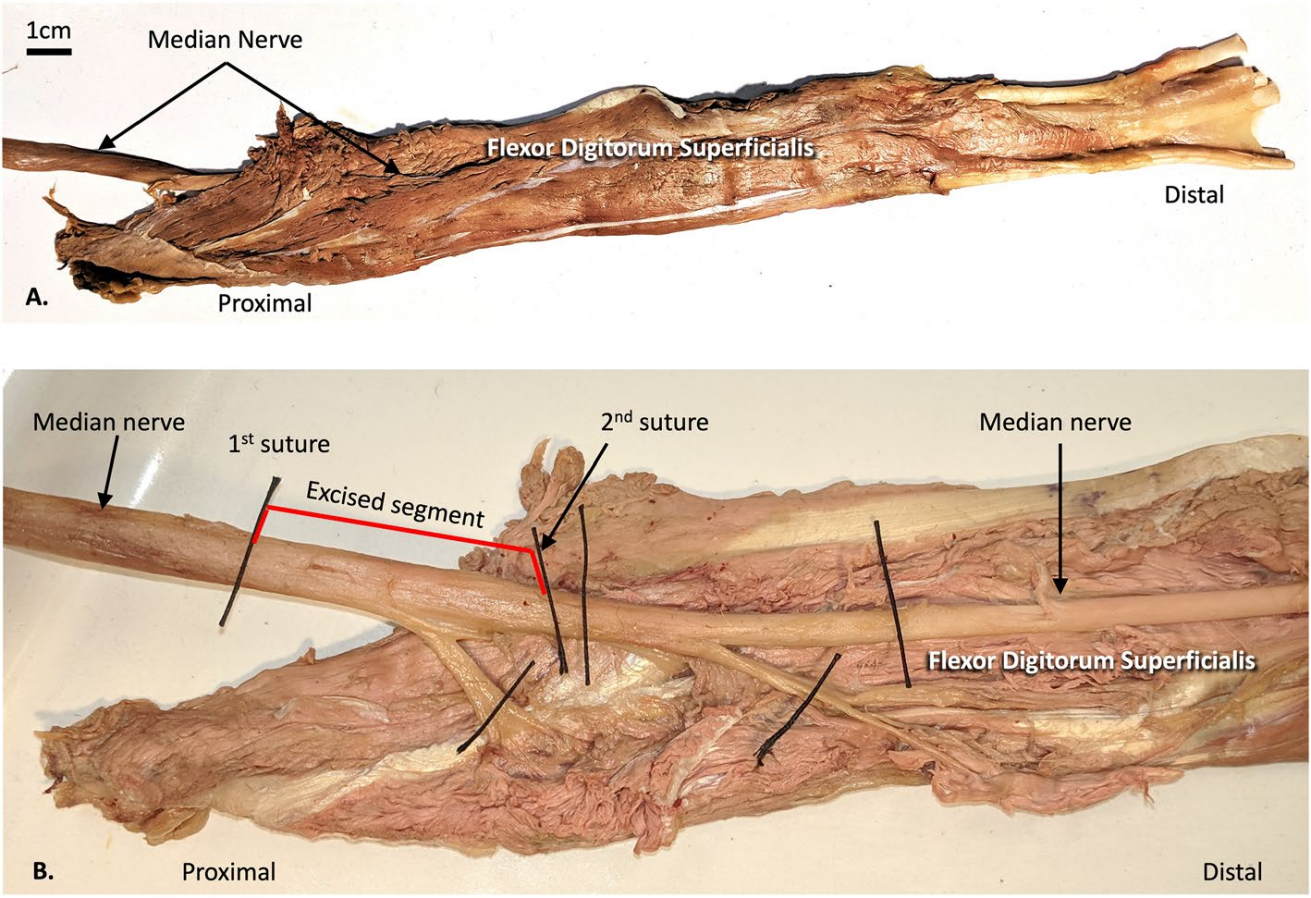
Cadaveric dissection of median nerve and flexor digitorum superficialis muscle. **A** Overview. **B** Excised segment of median nerve. Image adapted from [20] with permission

A nerve sample collected in a previous study [20] was used to generate the FE model used in this investigation. A 7 cm segment of the median nerve entering flexor digitorum superficialis along with the entire muscle belly was removed from the specimen (Fig. 2A). A 5 cm extramuscular segment of the median nerve was excised between the first and second suture indicated in Fig. 2B. The next step was to perform histologic sectioning of the excised segment of the median nerve (Centre for Phenogenomics in Toronto, Ontario). The nerve segment was transversely sectioned into six equal parts and fixed in formalin. Each part was embedded in paraffin and sectioned into 5 μm-thick slices, each 250 μm apart. The sections were stained with anti-neurofilament antibody IHC. An Olympus VS-120 whole slide scanning system was used to acquire high-resolution (5183 pixels per inch) images.

### Nerve FEM

**Fig. 3:**
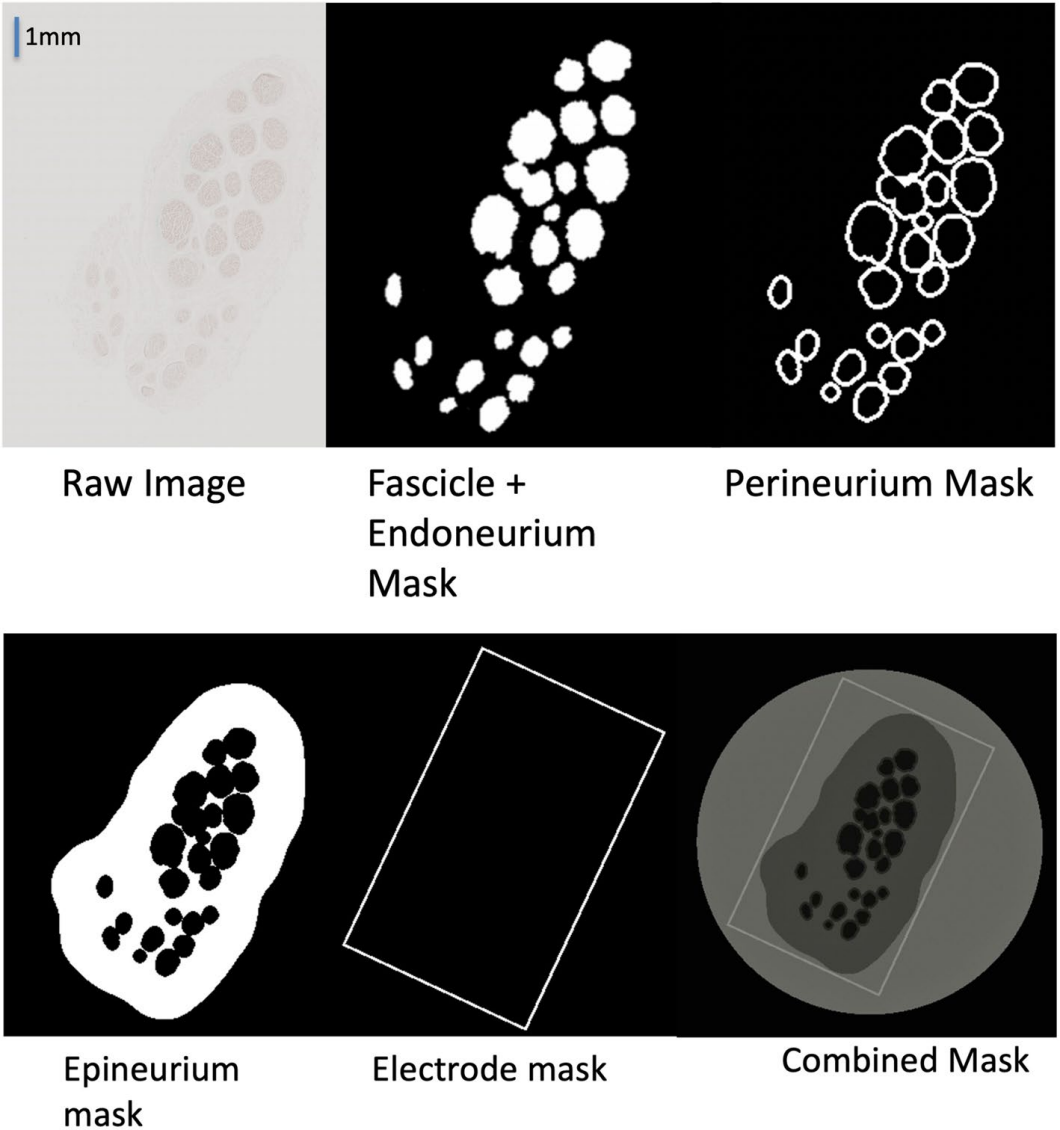
Material layer isolation. Raw image (top left), endoneurium mask (top middle), perineurium mask (top right), epineurium mask (bottom left), electrode mask (bottom middle), and combined mask (bottom right)

Starting from a single IHC image, masks for the tissue and material layers were defined as detailed below and are illustrated in Fig. 3. The slice was chosen fitting three criteria: proximal to branching resulting in high fascicle density, clear boundaries visible on fascicle and surrounding tissues, singular nerve segment without branches or gaps visible in cross section. The resulting cross-sectional data were extruded to obtain a 3 cm-long 3D grayscale image where each type of material (nerve tissue, saline, and electrode) was assigned to its own layer.

#### Endoneurium

The endoneurium of the nerve cross section was detected using MATLAB (MathWorks, Natick, USA) image processing libraries and Otsu’s method to detect the boundary edges of the fascicles from the IHC images [21]. Smoothing and sizing constraints were used to isolate fascicles and allow for meshing in later steps. A minimum size constraint was placed to prevent artifacts (blood vessels, imaging artifacts, or circular shapes in other areas of the connective tissue) from incorrectly being identified as a fascicle. These boundaries were filled, and masks of the fascicles were generated.

#### Perineurium

The perineurium layer was approximated using an average thickness of perineurium tissue in human median nerves [22]. The perineurium tissue was approximated by dilating the endoneurium mask by 3% and then connecting any overlapping regions into one singular area in the mask. The endoneurium mask was then subtracted to isolate the perineurium tissue.

#### Epineurium

The epineurium layer was masked by dilating the perineurium mask until the average thickness of the epineurium mask matched the original IHC image. Due to the poor quality of the endoneurium IHC image, this method was found to match the IHC reference image more closely than those generated through thresholding as outlined in [21]. The perineurium and endoneurium masks were then subtracted from the layer to isolate the epineurium tissue.

#### Electrode

The outer and inner dimensions of the electrode were defined following the dimensions of a FINE [23, 24]. The mask of the electrode was created by defining a hollow rectangle with electrode thickness matching the FINE with the length and width scaled to fit the modeled nerve cross section (‘short side’ length: 4.9 mm, ‘long side’ width: 9.0 mm, height: 20 mm, and material thickness: 0.65 mm). Due to the oblong shape of the nerve segment chosen as the basis for the model, a rectangular FINE electrode was selected for the simulations rather than a cylindrical nerve cuff. This configuration consisted of seven “layers” of contacts running axially along the nerve segment.

Each “layer” was a rectangular shape with two long and two short sides. Contacts were simulated to be on the two long edges of the rectangular cross section, four contacts equally spaced per edge. The inter-contact distance along one edge of a layer was 2.25 mm, and the inter-layer distance was 2.86 mm. The FINE electrode was modeled around the originally imaged cross section of the median nerve without any additional deformations of the nerve that may happen in practice during the application of the electrode. During in vivo application, the FINE is designed to deform the nerve to fit the cross section of the electrode. This allows for greater geometric separation between fascicles in the cross-sectional plane, improving recording selectivity. Due to a lack of literature on the expected migration of fascicles in this type of application, as well as the already elongated shape of the nerve in our sample, the original cross section of the nerve was maintained. This was done to retain the accuracy of the modeling of the tissue structures within the nerve segment and to act as a “worst case” scenario from a signal selectivity perspective.

#### Saline

The saline layer was modeled to be a cylinder of diameter 11 mm, approximately 20% wider than the long edge of the electrode, to prevent boundary effects in the electrical model of the nerve section [10].

### Computational model generation

**Fig. 4:**
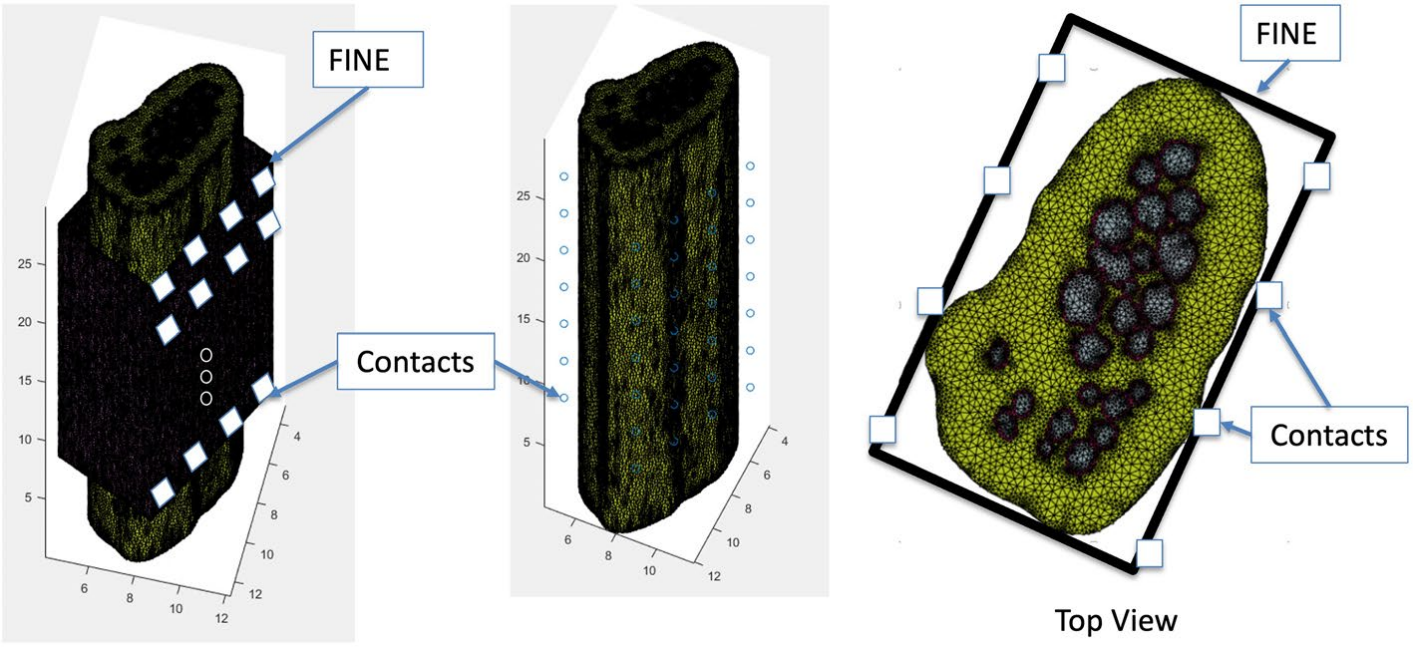
Finite-element mesh: isometric view with transparent saline layer (left), isometric view with transparent electrode and saline layers (middle), and cross-sectional view at center of mesh along *z*-axis and transparent saline layer (right)

Once the 3D geometry of the model had been created as described above, a tetrahedral mesh (Fig. 4) was generated using iso2mesh, an MATLAB-based mesh generator [25].

#### Finite-element model

The 3D mesh of the nerve segment was used to generate a computational model that simulated the propagation of the AP through the nerve and output the simulated recordings at each of the electrical contacts in the nerve cuff. Inter-node distances of < 0.1 mm or smaller were used to ensure that there was sufficient resolution to characterize the current path through the mesh. To refine the mesh design, larger mesh element sizes were used in the saline layer to allow for finer mesh resolution in the connective tissues, specifically the perineurium. Within the nerve, the mesh density was set to be sufficiently high, such that there were several elements of thickness for each tissue layer (i.e., no tissue layer was traversed at any point within the mesh with a single element in any direction). This was done to model the impact of each tissue layer on the simulated current pathways while retaining a small enough mesh to meet memory limitations of both SCIRun and MATLAB. The electrical model that was used to generate simulated APs was created using the process described by Weinstein et al. [26] and used the conductivity values in Table 1, drawn from [27, 28]. The relationship between the contact voltages and dipole sources located at the mesh elements is shown in Eq. 1

**Table 1:** Material conductivities

Material type	Conductivity (S/m)
Endoneurium (longitudinal)	0.5710
Endoneurium (transverse)	0.0826
Perineurium	0.0021
Epineurium	0.0826
Saline	2
Cuff	1 × 10^–7^

1 $$D=LJ.$$

The recording matrix $D$ is a matrix representation of the voltage values across all 56 contacts over $T$ timesteps of the simulation. The simulated action potential is represented in source matrix $J$ as a set of voltages across all elements in the mesh over $T$ timesteps in the simulation. The transformation from the voltages in the mesh to the voltages at the electrode contacts is done using leadfield matrix $L.$ The leadfield matrix is an *M* x *N* matrix used to map neural activities at different locations within the nerve to recordings at the nerve cuff contact sites. Briefly, the leadfield matrix was generated using current source–sink pairs between contacts in the electrode, and then applying the reciprocity principle to obtain measured voltages at the electrode contacts in response to a dipole current source in any given mesh element, as outlined in detail in [26]. The leadfield matrix was generated using FE analysis to solve the forward problem using SCIRun (NIH/NIGMS Center for Integrative Biomedical Computing, USA)—a software used to generate realistic bioelectric models [29]. The leadfield matrix generated in SCIRun was imported into MATLAB and converted to a tripolar reference using the average of the contacts in the outer rings. The resulting leadfield was then used to generate the simulated dataset for training and evaluating the CNN responsible for nCAP classification.

### Action potential simulation

One thousand ideal, noiseless nCAPs of 100 time samples at a sampling rate of 30 kHz each were generated with slight variation in signal amplitudes and offsets. Each simulated nCAP used a waveform randomly sampled from this set. The waveform was then mapped to mesh elements based on the cross section within the nerve, the conduction velocity (60 m/s), and the spacing between the nodes of Ranvier to create the source matrix *J,* similarly to the process described in [16, 30]. The neural source data in *J* were then multiplied with the leadfield matrix to obtain the simulated recordings from the 56 electrode array [26].

The noiseless time-series output from the simulation then had white noise added to it. The variance of the noise added was determined using a ratio of the maximum absolute value across all channels, outlined in Eq. 2

2 $$\mathrm{var}\left(\mathrm{noise}\right)=n\times \mathrm{max}\left|\mathrm{noiseless~} \mathrm{signal~} \mathrm{across~} \mathrm{all~} \mathrm{channels}\right|$$

**Fig. 5:**
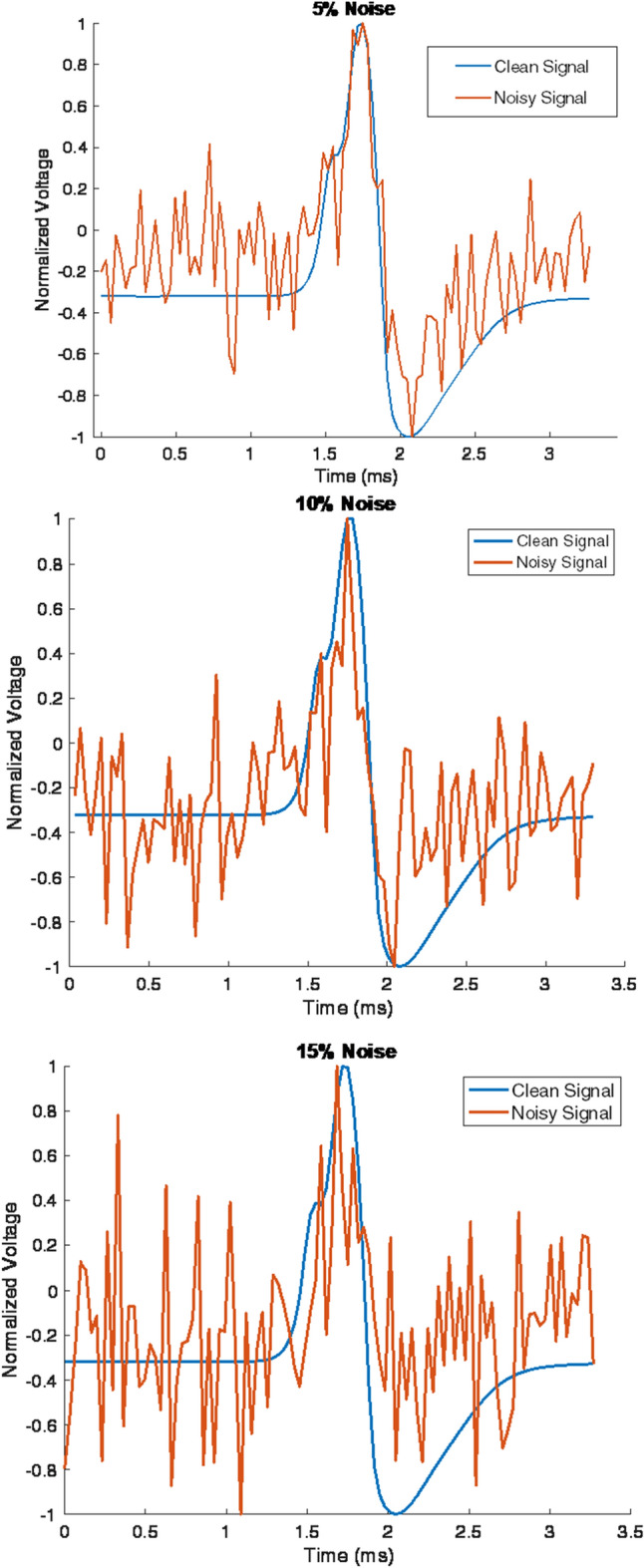
5% (Top), 10% (middle), and 15% (bottom) noise samples

The noisy data were compared to in vivo rat data to select a scaling factor n that produced an appropriate SNR. The average post-filtering noise (no spike train) and signal (spike train) voltage values from previous rat experiments were used to determine the magnitude of the simulated noise. The ratio of voltages was replicated in simulation to ensure that the simulated instrument noise was within the range observed in vivo. Examples of the three noise levels investigated are shown in Fig. 5.

To prevent the CNN from learning absolute voltages rather than spatiotemporal patterns, all signals were normalized to the maximum absolute value of the voltage across all channels. This was done to ensure that classification was based on relative patterns between channel, rather than on absolute signal amplitudes.

The data was then processed into 56 × 100 spatiotemporal signatures to train and test the CNNs.

### Spatiotemporal signatures

**Fig. 6:**
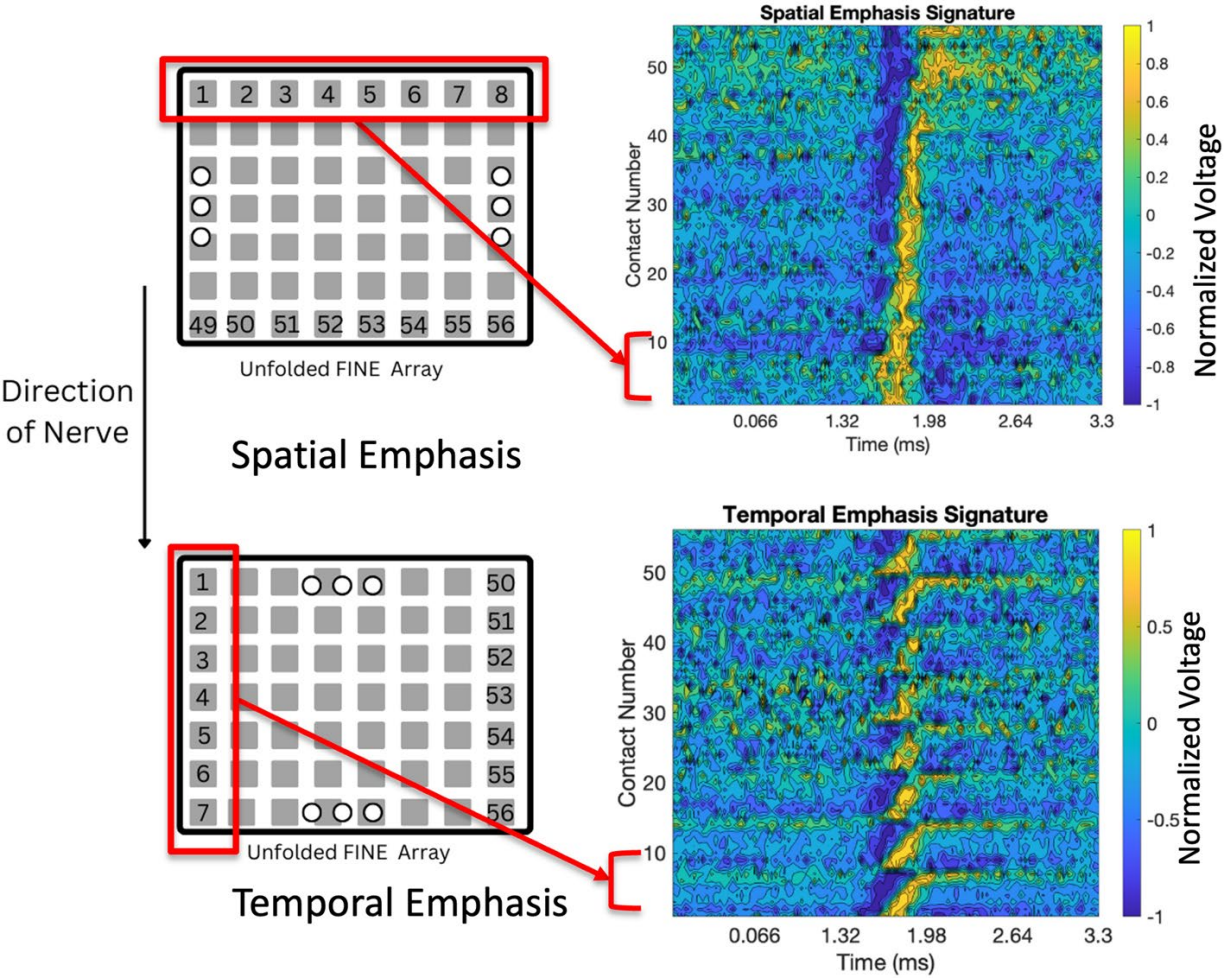
Spatial (above) vs. temporal (below) emphasis representations of electrode array signals

Spatiotemporal signatures were generated for each simulated nCAP. “Spatial emphasis” (SE) signatures were created by grouping contacts along each rectangular layer, while “temporal-emphasis” (TE) signatures were created by grouping contacts axially along the nerve [14]. The electrode configurations and the resulting spatiotemporal signatures can be found in Fig. 6.

Spatiotemporal signatures of the electrode signals are represented with time on the *x*-axis, contact number of the *y*-axis, and the value of each pixel denoting the recorded voltage. These were then used as inputs to the CNN [14].

### CNN implementation

ESCAPE-NET is a custom CNN built along a classical architecture. The performance of the ESCAPE framework was evaluated with ESCAPE-NET as well as with ResNet-50 and MobileNetV2, which have been shown to have high accuracy when used with image sets [31–33]. These architectures were chosen based on three criteria: network complexity, training set size requirements, and top-1% accuracy on the MNIST dataset. Future application of this technology will likely require on-chip and real-time application; therefore, networks of smaller complexity were given higher priorities. High accuracy is necessary due to the potential impacts of misclassifying the neural signals (potentially resulting in difficult-to-use prosthetics if applied as a control system). The goal of this research was to empower a future solution that may be applied to low-power processing modules, and as a result, required networks of relatively low depth and training requirements. As a result, the entire training and test set was capped at 30,000 samples, even though simulated data can allow for much larger datasets. The network size was also capped at networks below 100 million parameters.

ESCAPE-NET, with 92 million parameters, ResNet-50, with 23 million parameters, and MobileNet-V2, with 3.4 million parameters were determined to be suitable networks that represent deep architectures of varying orders of magnitude. This allowed for an investigation into the effect of network complexity on classifying PNI data.

ESCAPE-NET uses a 2-stream architecture taking SE and TE signatures as inputs, and then concatenating the extracted features [14] The alternative CNNs (ResNet-50, MobileNet-V2) were tested by replacing the base CNN in each stream of the multi-stage architecture with the new network under investigation.

#### ESCAPE-NET implementation

Each stream of the ESCAPE-NET architecture follows a common CNN architecture using convolutional layers, pooling layers, and a fully connected layer [14]. The version of ESCAPE-NET used in this study was an updated version previously introduced in [15], which included additional dropout layers to improve generalizability. ESCAPE-NET was implemented using the Keras wrapper of TensorFlow libraries. ESCAPE-NET was implemented with a learning rate of 1e-3, decay of 1e-6, momentum of 1e-9, and an early stopping criterion of 15 epochs of no improvement in the validation loss.

#### ResNet-50 implementation

ResNet is one of the most widely used CNN architectures, for both image and non-image datasets. ResNet employs a multi-branch architecture and its variants have been shown to outperform most other networks with similar complexity for image classification [31, 33]. The ResNet implementation used in this study was the ResNet-50 Keras package. This was chosen due to it being a widely used ResNet implementation (stable package support and high likelihood of use in similar future studies) that met the requirements of the CNNs of interest in this study. ResNet-50 was implemented with a learning rate of 1e-3, decay of 1e-6, momentum of 1e-9, and an early stopping criterion of 40 epochs of no improvement in the validation loss. Learning rates of 1e-2 and 1e-4 were also investigated and found not to provide improved performance.

#### MobileNet-V2 implementation

MobileNet-V2 is a network that maximizes skipped connection blocks to improve its robustness toward a vanishing gradient upon backpropagation and optimization steps to minimize computational and memory requirements [34]. The MobileNet implementation used for this study was the Keras package MobileNet-V2. This was chosen due to it being the latest stable and widely used implementation of Mobile Net. MobileNet-V2 was implemented with a learning rate of 1e-3, decay of 1e-6, momentum of 1e-9, and an early stopping criterion of 40 epochs of no improvement in the validation loss. Learning rates of 1e-2 and 1e-4 were also investigated and found not to provide improved performance.

### Performance evaluation

The evaluation was based on the ability to correctly identify the fascicle in which an nCAP originated. Classification accuracies were used to evaluate the performance of the CNNs. Due to the balanced nature of the dataset, the macro- and micro-F1-scores did not provide any additional insight to the performance of the network over accuracy and thus are not reported here.

Each network was trained and evaluated with simulated data using a threefold cross-validation method for each number of classes. The scenarios consisted of 3, 5, 7, and 10 active fascicles, with 5%, 10%, and 15% noise, for a total of 12 test conditions. Ten complete training/test datasets were generated for each of these conditions for a total of 120 experiments per network. All datasets consisted of 30,000 nCAPs, balanced among the number of classes for that scenario. Each dataset (for example each of the datasets of the 10 fascicles and 15% noise scenario) was used for threefold cross-validation, each time including 20,000 simulated nCAPs for the training set and 10,000 simulated nCAPs for the test set. The size of the training sets are consistent with our previous in vivo work, in which several thousand nCAPs could be acquired through repeated sensory stimuli over the course of a few minutes [14].

The effect of the number of classes was investigated by varying the number of possible sources in the simulated recordings. Each neural pathway included in a simulation was a separate class in the classification task. The nerve segment chosen for simulation contained 17 possible fascicles as nCAP sources. The steps for evaluating each case were as follows:

1. Determine the number of classes to be investigated (c classes)
2. Pick c fascicles at random from the 17 possible pathways to act as the neural pathways of interest.
  - Cross-sectional location (node of nCAP origin in the mesh) within the selected fascicle was picked at random for each nCAP to add variability to simulated recordings from each fascicle.
3. Develop ten datasets for each value of c, each with a different random selection of fascicles.
4. Perform CNN training and testing for each of the ten datasets for each c-class case.
  - Average accuracy for each test scenario was used as evaluation metrics, reported as mean ± standard deviation across the ten repetitions.

### Statistical analysis

A two-way analysis of variance (ANOVA) was used to determine the influence of the number of classes and the noise level on the classification accuracy, as well as interactions between the number of classes and the noise level. The two-way ANOVA was performed using the MATLAB statistics package.

## Data Availability

Study materials can be shared upon reasonable request to the authors.

## References

[1] Dillingham TR, Pezzin LE, MacKenzie EJ (2002). Limb amputation and limb deficiency: epidemiology and recent trends in the United States. South Med J.

[2] Varma P, Stineman MG, Dillingham TR (2014). Epidemiology of limb loss. Phys Med Rehabil Clin N Am.

[3] Ziegler-Graham K, MacKenzie EJ, Ephraim PL, Travison TG, Brookmeyer R (2008). Estimating the prevalence of limb loss in the United States: 2005 to 2050. Arch Phys Med Rehabil.

[4] Vu PP, Vaskov AK, Lee C, Jillala RR, Wallace DM, Davis AJ (2023). Long-term upper-extremity prosthetic control using regenerative peripheral nerve interfaces and implanted EMG electrodes. J Neural Eng.

[5] Vu PP, Vaskov AK, Irwin ZT, Henning PT, Lueders DR, Laidlaw AT (2020). A regenerative peripheral nerve interface allows real-time control of an artificial hand in upper limb amputees. Sci Transl Med..

[6] Brill NA, Tyler DJ (2017). Quantification of human upper extremity nerves and fascicular anatomy. Muscle Nerve.

[7] Metcalfe B, Nielsen T, Taylor J. Velocity selective recording: a demonstration of effectiveness on the vagus nerve in pig. In: Proceedings of the annual international conference of the IEEE engineering in medicine and biology society, EMBS. 2018. 10.1109/EMBC.2018.8512991 30440281

[8] Koh RGL, Nachman AI, Zariffa J (2019). Classification of naturally evoked compound action potentials in peripheral nerve spatiotemporal recordings. Sci Rep.

[9] Hwang YCE, Long L, Filho JS, Genov R, Zariffa J. Closed-loop control of functional electrical stimulation using a selectively recording and bidirectional nerve cuff interface. 2023. bioRxiv. 2023–06. 10.1109/TNSRE.2024.3355063 38231810

[10] Yoo PB, Durand DM. Selective fascicular recording of the hypoglossal nerve using a multi-contact nerve cuff electrode. In: Annual international conference of the IEEE engineering in medicine and biology—Proceedings. 2003.

[11] Ghafoor U, Kim S, Hong KS (2017). Selectivity and longevity of peripheral-nerve and machine interfaces: a review. Front Neurorobot.

[12] del Valle J, Navarro X (2013). Interfaces with the peripheral nerve for the control of neuroprostheses. Tissue engineering of the peripheral nerve—biomaterials and physical therapy.

[13] Christie BP, Freeberg M, Memberg WD, Pinault GJC, Hoyen HA, Tyler DJ (2017). Long-term stability of stimulating spiral nerve cuff electrodes on human peripheral nerves. J Neuroeng Rehabil.

[14] Koh RGL, Balas M, Nachman AI, Zariffa J (2020). Selective peripheral nerve recordings from nerve cuff electrodes using convolutional neural networks. J Neural Eng.

[15] Koh RGL, Jabban L, Fukushi M, Adeyinka IC, Zariffa J, Metcalfe B. A comparison of extraneural approaches for selective recording in the peripheral nervous system. In: Proceedings of the annual international conference of the IEEE Engineering in Medicine and Biology Society, EMBS. 2022. 10.1109/EMBC48229.2022.9871727 36086428

[16] Sammut S, Koh RGL, Zariffa J (2021). Compensation strategies for bioelectric signal changes in chronic selective nerve cuff recordings: a simulation study. Sensors (Switzerland)..

[17] Yan L, Guo Y, Qi J, Zhu Q, Gu L, Zheng C (2017). Iodine and freeze-drying enhanced high-resolution MicroCT imaging for reconstructing 3D intraneural topography of human peripheral nerve fascicles. J Neurosci Methods.

[18] Thompson N, Ravagli E, Mastitskaya S, Iacoviello F, Aristovich K, Perkins J (2020). MicroCT optimisation for imaging fascicular anatomy in peripheral nerves. J Neurosci Methods.

[19] Pedreira C, Martinez J, Ison MJ, Quian QR (2012). How many neurons can we see with current spike sorting algorithms?. J Neurosci Methods.

[20] Tovbis D, Agur A, Mogk JP, Zariffa J. Automatic three-dimensional reconstruction of fascicles in peripheral nerves from histological images. PLoS ONE. 2020;15(5): e0233028. 10.1371/journal.pone.0233028 PMC7224505 32407341

[21] Otsu N (1996). A threshold selection method from gray-level histograms. IEEE Trans Syst Man Cybern.

[22] Grinberg Y, Schiefer MA, Tyler DJ, Gustafson KJ (2008). Fascicular perineurium thickness, size, and position affect model predictions of neural excitation. IEEE Trans Neural Syst Rehabil Eng.

[23] Tyler DJ, Durand DM (2002). Functionally selective peripheral nerve stimulation with a flat interface nerve electrode. IEEE Trans Neural Syst Rehabil Eng.

[24] Yoo PB, Durand DM (2005). Selective recording of the canine hypoglossal nerve using a multicontact flat interface nerve electrode. IEEE Trans Biomed Eng.

[25] QianQian F (2018). Iso2Mesh MATLAB ToolBox.

[26] Weinstein D, Zhukov L, Johnson C (2000). Lead-field bases for electroencephalography source imaging. Ann Biomed Eng.

[27] Raspopovic S, Petrini FM, Zelechowski M, Valle G (2017). Framework for the development of neuroprostheses: from basic understanding by sciatic and median nerves models to bionic legs and hands. Proc IEEE.

[28] Zariffa J, Popovic MR (2009). Localization of active pathways in peripheral nerves: a simulation study. IEEE Trans Neural Syst Rehabil Eng.

[29] MacLeod RS, Weinstein OM, Davison de St Germain J, Brooks DH, Johnson CR, Parker SG. SCIRun/BioPSE: integrated problem solving environment for bioelectric field problems and visualization. In: 2004 2nd IEEE international symposium on biomedical imaging: macro to nano (IEEE Cat No 04EX821). IEEE; p. 640–3.

[30] Koh RGL, Nachman AI, Zariffa J (2017). Use of spatiotemporal templates for pathway discrimination in peripheral nerve recordings: a simulation study. J Neural Eng.

[31] Bianco S, Cadene R, Celona L, Napoletano P (2018). Benchmark analysis of representative deep neural network architectures. IEEE Access..

[32] Canziani A, Paszke A, Culurciello E. An analysis of deep neural network models for practical applications. 2016. arxiv:1605.07678.

[33] Xie S, Girshick R, Dollár P, Tu Z, He K. Aggregated residual transformations for deep neural networks. In: Proceedings of the IEEE conference on computer vision and pattern recognition. 2017. p. 1492–500.

[34] Sandler M, Howard A, Zhu M, Zhmoginov A, Chen LC. MobileNetV2: inverted residuals and linear bottlenecks. In: Proceedings of the IEEE conference on computer vision and pattern recognition. 2018, p. 4510–20.

